# A Feedback Loop between Inflammation and Zn Uptake

**DOI:** 10.1371/journal.pone.0147146

**Published:** 2016-02-04

**Authors:** Paola Bonaventura, Aline Lamboux, Francis Albarède, Pierre Miossec

**Affiliations:** 1 Department of Immunology and Rheumatology, Immunogenomics and inflammation research Unit EA 4130, University of Lyon, Edouard Herriot Hospital, Lyon, 69437 France; 2 Geology Laboratory–Department of Earth Sciences, Ecole Normale Supérieure de Lyon and CNRS Lyon, Lyon, 69364 France; French National Centre for Scientific Research, FRANCE

## Abstract

**Objective:**

Zinc (Zn) has major effects on the immune system and inflammation is associated with systemic Zn deficiency. The aim of this work was to investigate how inflammation modifies Zn metabolism at the cellular level. Rheumatoid arthritis (RA) synoviocytes exposed to cytokines were used as a model of chronic inflammation. Osteoarthritis (OA) synoviocytes were used as control.

**Methods:**

Zn levels were measured in medium and inside cells by Induced Coupled Plasma-Mass Spectrometry (ICP-MS), in the presence of minute quantities of stable spike ^70^Zn isotope and the addition or not of the pro-inflammatory cytokines interleukin-17 (IL-17) and tumor necrosis factor alpha (TNF-α). Gene expression of ZIP-8 importer, ZnT1 exporter and the homeostasis regulators metallothioneins (MTs) was evaluated after pre-exposure to cytokines, with or without exogenous Zn addition at increasing concentrations. IL-6 production was used as a marker of inflammation and measured by ELISA.

**Results:**

Exposure to IL-17 and TNF-α enhanced expression of the Zn-importer ZIP-8, regardless of the concentration of Zn in the culture medium. In contrast, the expression of the Zn-exporter ZnT1 and of the MTs was primarily dependent on Zn levels. Addition of Zn also increased the production of IL-6, thus further stimulating the inflammatory response.

**Conclusion:**

IL-17/TNF-mediated inflammation enhanced the intracellular Zn uptake by synoviocytes, further increasing inflammation. These observations document the existence of a feedback loop between inflammation and Zn uptake. Based on these results, a mathematical model was developed to represent the cytokine-mediated Zn homeostasis alterations.

## Introduction

Zinc (Zn) is an essential trace element, which is ubiquitous at low levels in the environment and required for human health [1]. Low levels of Zn contribute to the immune defects associated with malnutrition. Conversely, exposure to high doses of Zn e.g., in the context of mining or manufacturing activities, has toxic effects with adverse consequences on the immune system [2].

At the cellular level, Zn is involved in cell metabolism, survival, and immune response mechanisms [3,4]. Zn is a component of numerous proteins including the metallo-proteases (MMPs), involved in matrix remodeling [5], the carbonic anhydrases in cell respiration [6] and the TNF-α converting enzyme (TACE) in the proteolytic release of cytokines, such as TNF-α [7]. As part of these important mechanisms for cell survival and inflammatory response, Zn has a major role in inflammation.

Zinc transport through cells is controlled by the SLC39 importers (Zrt-Irt-Proteins, ZIPs 1 to 14), by the SLC30 exporters (Zn-Transporters, ZnTs 1–10, with ZnT1 as only membrane exporter) and the homeostasis regulators metallothioneins (MT-1 and -2). MTs are low molecular weight, cysteine-rich, heavy metal-binding proteins. Binding Zn, MTs act as a Zn pool in cells and modulate the immune system [8], as well as the response to stress conditions, including the exposure to heavy metals.

Although Zn importance in the pathogenesis of chronic inflammatory diseases is fully appreciated systemically, its implication at the cellular level is not well known [4,9–12]. Synovial fibroblasts, isolated from rheumatoid arthritis (RA) patient biopsies, referred to as synoviocytes, were selected as a model of chronic inflammation, illustrating how stromal cells could contribute to the persistence of inflammation through the acquisition of defective apoptosis. Synoviocytes from osteoarthritis patients (OA) were used as control, as representing a less inflammatory state of the synovium.

Fibroblasts express some of the Zn transporters (ZIP-8 [13], MTs-1 [14]) and respond rapidly to inflammation [15]. They are active participants in the immune response, interacting with, promoting the activation of T cells [16,17], and thereby influencing the switch from acute to chronic inflammation [18]. In chronic inflammation, an infiltration of immune cells at the disease site leads to self-sustained production of pro-inflammatory cytokines, notably interleukin-17 (IL-17) and tumor necrosis factor alpha (TNF-α). These two cytokines often act synergistically on synoviocytes, inducing high IL-6 production, which in turn triggers the activation of inflammatory cells (B cells, T cells, synoviocytes, and osteoclasts) and their highly aggressive phenotype [19]. IL-6 has been shown to be a central pro-inflammatory cytokine involved in RA development and IL-6 blockade with a humanized anti-IL-6 receptor antibody has proved its efficacy either as monotherapy or in combination with disease-modifying anti-rheumatic drugs [20].

The aim of our work was to investigate how the combination of IL-17 and TNF-α can modify cellular Zn homeostasis in synoviocytes, eventually contributing to chronicity. The use of minute quantities of stable ^70^Zn provided a novel perspective to study the metal kinetics in cells. A model of diffusion-controlled Zn transport was developed to account for the observed variations of Zn flux through cells under inflammatory vs. normal conditions.

## Materials and Methods

### Primary cell isolation, cell culture, and experimental design

Synoviocytes were grown from fresh synovial tissue samples aseptically isolated from RA and osteoarthritis (OA) patients’ joints. The RA patients fulfilled the American College of Rheumatology criteria for RA [21]. All patients signed an informed consent form and the study was approved by the ethics committee of the hospitals of Lyon. The synovial tissue, minced in small pieces, was allowed to adhere to plastic plates and maintained in DMEM medium (Eurobio, Courtaboeuf, FR) supplemented with 10% FBS (Life Technologies by Thermo Fischer scientific, Grand Island, NY, USA), 2% Penicillin-Streptomycin, 1% L-glutamine and 1% Amphotericin B (all Eurobio) until cells reach 90% confluence. Synoviocytes were used between the fourth and ninth passages to ensure the cell specificity. All data are the results of at least three separate experiments (n = 3) and patients.

### Cytokines and Zn exposures

Synoviocytes were pre-exposed overnight to a combination of IL-17A at 50 ng/ml (R&D systems, Minneapolis, MN, USA) and TNF-α at 0.5 ng/ml (R&D systems), prior to Zn exposure. Normal medium contains between 0.2 and 0.3 ppm of Zn. Zn was added in nitrate form to increase bulk concentration to 0.9 ppm or 3.6 ppm of Zn. In order to assess the extent of exchange between the cells and the ambient medium, negligible amounts of isotope ^70^Zn were added (10 ppb)

### Zinc kinetics and K_D_ by ICP-MS

Synoviocytes (5*10^5^) were cultured for 5 days with and without cytokines. Supernatants were collected at 6, 48, 96, and 120 hours, while cells were collected, and counted at day 5. Samples were mineralized with HNO_3_ 0.5N plus H_2_O_2_ (15–20%) at 100°C and re-dissolved in a 5% HNO_3_ solution in deionized water. Metal ions in the samples were measured on a single collector ICP-MS platform ELEMENT 2 (Thermo Finnigan, Ringoes, NJ, USA). Zn found in cells was adjusted to the number of cells. Zinc fractionation constant (K_D_) between cells and medium was calculated as K_D_ = (^64^Zn/^70^Zn)_cells_/(^64^Zn/^70^Zn)_medium_.

### Zinc-transporter quantitative RT-PCR analysis

Synoviocytes were exposed or not to cytokines overnight followed or not by a six-hour Zn exposure. Total RNA was extracted using the RNeasy Mini Kit (Qiagen^®^, Hilden, GE), quantified with the Quant-it kit assay (Invitrogen^™^ by Thermo Fisher Scientific, Grand Island, NY, USA) and RNA was reverse-transcribed with the QuantiTect Reverse Transcription Kit (Qiagen^®^, GE). PCR amplification was performed on a CFX96 Real-Time PCR Detection System (BioRad, Hercules, USA) using the QuantiFast SYBR Green PCR Kit (Qiagen^®^, GE). The expression of the genes was normalized to the expression of GAPDH.

### Measurement of the production of the pro-inflammatory cytokine IL-6

IL-6 production was quantified with standard ELISA kit (R&D system, San Diego, CA, USA) in the supernatants of synoviocytes exposed to cytokines and Zn at day 1, 5 and 8.

#### Statistical analysis and modeling

Statistical significance of changes in data (mean ± standard error of the mean, SEM) was determined by GraphPad Prism^™^ using nonparametric statistical methods. A two-way ANOVA test was used in the case of exposure to both Zn and cytokines. A Mann-Whitney test or Wilcoxon matched-pairs signed rank test were used in the case of single exposure. p-values inferior or equal to 0.05 were considered statistically significant. A model of diffusion-controlled Zn transport was constructed to describe the variation of Zn flux through the cells with or without IL-17 and TNF-α addition.

## Results

### Zinc uptake is increased by the combination of IL-17 and TNF-α

**Fig 1:**
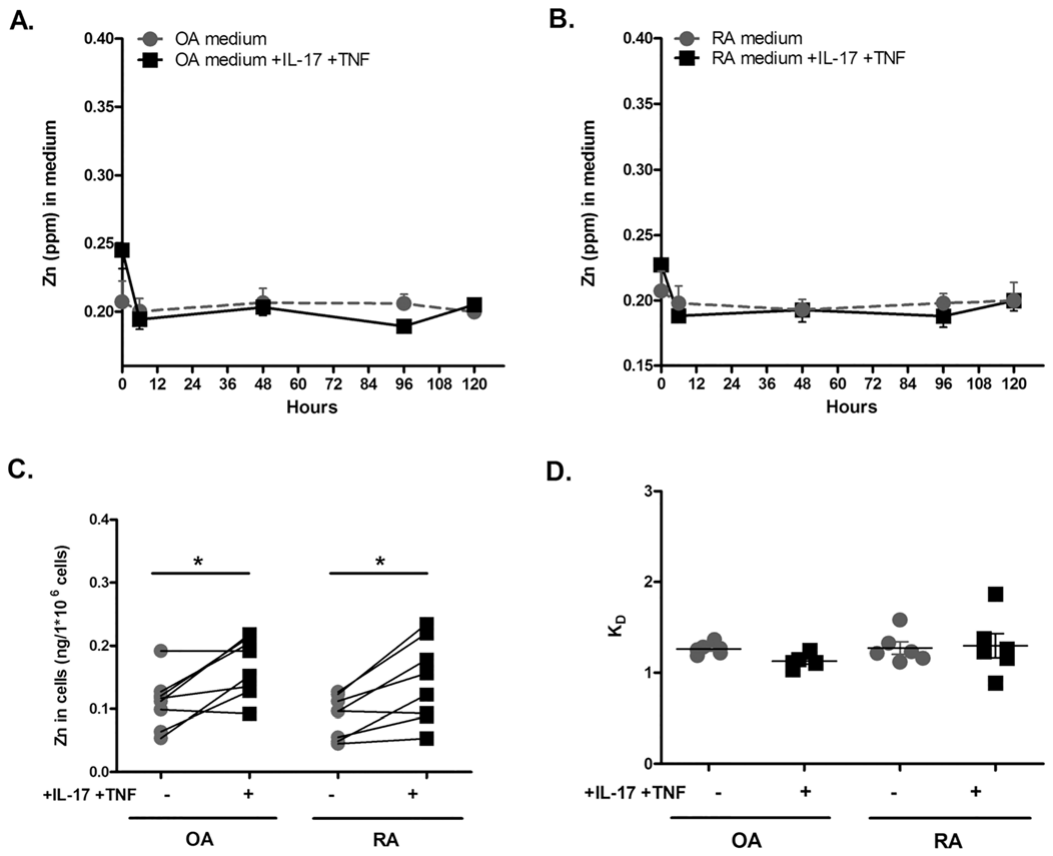
Zinc is more absorbed by synoviocytes under inflammatory conditions. (A) Zn concentration in the OA synoviocyte culture experiment as a function of time. Dashed line: control experiment with no cytokine addition. Solid line: cytokines added to the culture medium. (B) Zn concentration in the RA synoviocyte culture experiment as a function of time. Dashed and solid line as in A. (C) Zn uptake calculated from Zn concentration measured in the cells expressed in ng per 10^6^ cells at time t = 120 h. (D) K_D_ = (^66^Zn/^70^Zn)_cells_/(^66^Zn/^70^Zn)_medium_ at 120h after addition of ultra-trace amounts of ^70^Zn. At equilibrium, K_D_ =. Data are the mean of at least three independent experiments. Data are presented as mean ± SEM; * p<0.05.

To evaluate the effect of inflammation on the homeostasis of Zn, Zn content was measured in culture medium and intracellularly in OA and RA synoviocytes exposed or not to the combination of IL-17 and TNF-α for up to 120 hours. A transient Zn decrease in medium was seen during the first 6 hours of culture in both cell types (Fig 1A and 1B; S1 Table). The effect of cytokine addition was not measurable in the medium, while there was evidence of Zn accumulation in cells after 120 hours (Fig 1C; S1 Table). In both types of synoviocytes, the accumulation of Zn was increased by the combination of IL-17 and TNF-α in comparison to the control situation (Fig 1C, OA synoviocytes: 0.11 ± 0.01 ng/10^6^ cells without cytokines vs. 0.17 ± 0.02 ng/10^6^ cells with cytokines, p = 0.04; RA synoviocytes: 0.09 ± 0.01 ng/10^6^ cells without cytokines vs. 0.14 ± 0.02 ng/10^6^ cells with cytokines, p = 0.02; S1 Table). RA synoviocytes had a tendency for a more pronounced heterogeneity between cell lines from different patients (Fig 1C).

Zn K_D_ represents the isotopic fractionation constant between cells and medium (see materials and methods). Values of K_D_ of ~1 indicate an isotopic exchange equilibrium, whereas K_D_>1 indicates that the exchange did not still reach the equilibrium. K_D_s of Zn in both types of synoviocytes were ~1, indicating a nearly perfect Zn isotopic equilibrium between cellular intake and efflux (Fig 1D, see the analytical model; S1 Table). K_D_s in RA synoviocytes exposed to pro-inflammatory cytokines reproduced the heterogeneity already observed with the cell content (Fig 1C).

### TNF-α and IL-17 combination enhances zinc importer gene expression and modifies other trafficking molecule gene expression

**Fig 2:**
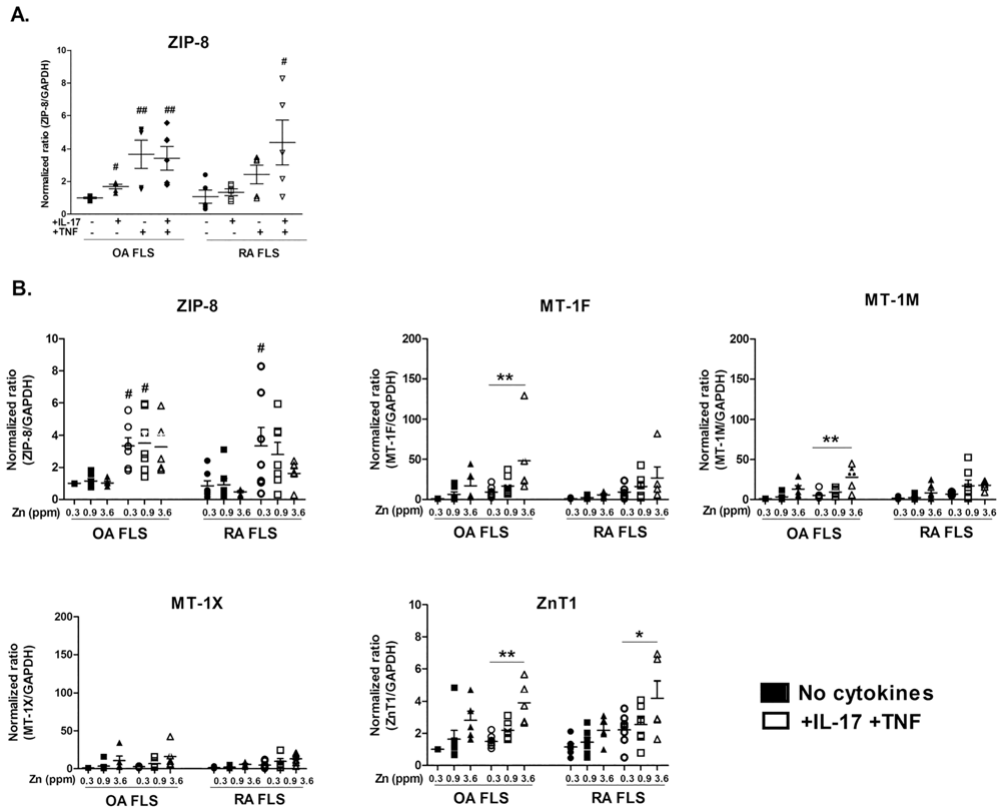
TNF-α and IL-17 enhance zinc importer gene expression and modify other trafficking molecule gene expression. (A) The gene expression of the importer ZIP-8 was quantified after overnight exposure to IL-17A and TNF-α alone or in combination. (B) The gene expression of several Zn transporters was quantified by q-RT-PCR in synoviocytes under control conditions and in synoviocytes exposed overnight to a combination of recombinant IL-17A (50ng/mL) and TNF-α (0.5ng/mL) in the presence of increasing Zn concentrations.

To identify which mechanism increased Zn cellular content during inflammation, the expression of ZIP-8 importer was examined in synoviocytes exposed to IL-17 and TNF-α. The effect of IL-17 alone on ZIP-8 gene expression was limited in OA (Fig 2A, 1.00 ± 0.05 vs. 1.69+ ± 0.14, p<0.05) and absent in RA synoviocytes. In OA synoviocytes, TNF-α alone significantly enhanced the expression of ZIP-8 (1.00 ± 0.05 vs. 3.67 ± 0.85; p<0.01), but addition of both IL-17 and TNF-α had no further effect (Fig 2A). In RA synoviocytes, the presence of TNF-α alone was not sufficient to enhance ZIP-8 expression (Fig 2A, control 1.08 ± 0.40 vs. TNF-α alone 2.43 ± 0.57) but the combination of IL-17 and TNF-α enhanced ZIP-8 expression synergistically (Fig 2A, control: 1.08 ± 0.40 vs. combination of IL-17 and TNF-α: 4.38 ± 1.35; p<0.05) (S2 Table).

Once demonstrated the strong impact of the combination of IL-17 and TNF-α on RA synoviocytes, the expression of other Zn trafficking molecules was studied after exposure to both cytokines at different Zn concentrations.

#### Importer

The addition of Zn did not result in any change in ZIP-8 gene expression in OA synoviocytes, in the absence or presence of IL-17 and TNF-α. In contrast, in RA synoviocytes, Zn addition inhibited the effect of cytokines on ZIP-8 expression in a dose dependent fashion (Fig 2B; S2 Table).

#### Regulators

Addition of Zn to synoviocytes increased gene expression of MTs, the metal-binding proteins that regulates metal homeostasis. MTs expression was further enhanced with the combination of IL-17 and TNF-α (Fig 2B; S2 Table), while the presence of the highest concentration of Zn affected less the expression of MTs in RA than in OA synoviocytes (i.e. in the presence of 3.6 ppm of Zn, MT-1F was 5.56 ± 1.49 in RA cells vs. 17.03 ± 8.50 in OA cells).

#### Exporter

The combination of cytokines had no significant effect on the expression of the exporter ZnT1 in both OA and RA synoviocytes. The expression of ZnT1 was slightly enhanced in proportion to Zn in both cell types (Fig 2B). Its expression was significantly increased only in the presence of the highest concentration of Zn (3.6 ppm) and the IL-17 and TNF-α combination in both OA and RA synoviocytes (Fig 2B, OA cells: +IL-17 and TNF-α: 1.5 ± 0.2 without Zn addition vs. 3.9 ± 0.6 with 3.6 ppm Zn addition, p<0.01; same conditions for RA cells +IL-17 and TNF-α: 2.3 ± 0.4 vs. 4.2 ± 1.1, p<0.05; S2 Table).

Results are normalized with GAPDH expression and are presented as fold changes compared to control. Data are the mean of at least three independent experiments. For data in which the effects of both cytokines and Zn are assessed, a two-way Anova test was used (A). For data for which only the effect of cytokines is assessed, a Mann-Whitney test was used (B). Data are presented as mean ± SEM; */# p<0.05, **/## p<0.01, # refers to the analysis of cytokine exposure and * refers to the analysis of Zinc exposure.

### Exposure to zinc and pro-inflammatory cytokines synergistically enhances IL-6 production by synoviocytes

**Fig 3:**
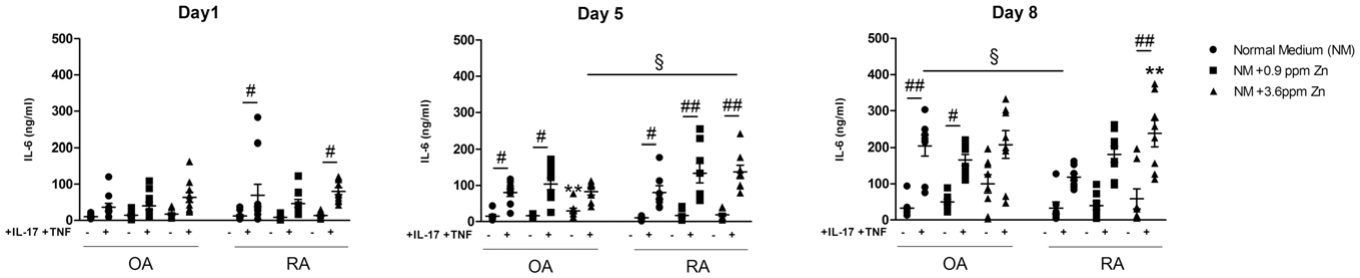
Exposure to Zn and pro-inflammatory cytokines synergistically affect IL-6 production in RA synoviocytes. OA and RA synoviocytes were treated overnight with IL-17A (50 ng/mL) and TNF-α (0.5 ng/mL) followed by the addition of different Zn concentrations (0.9 and 3.6 ppm). Supernatants were collected at day 1, 5 and 8. IL-6 production was quantified by ELISA. Statistical significance was assessed by a two-way Anova. Data are presented as mean ± SEM; # refers to the analysis of cytokine exposure, * to the analysis of Zinc exposure, § to the analysis of differences between OA and RA synoviocytes. */#/§ p<0.05, **/##/§§ p<0.01.

Zinc is known to regulate inflammation through the alteration of the immune system and systemic Zn deficiencies have been associated with several immune deficiencies and autoimmune diseases [4]. Therefore, the effect of Zn supplementation on the production of IL-6, the main pro-inflammatory cytokine secreted by fibroblasts, was evaluated at the cellular level in both inflammatory and non-inflammatory conditions. In agreement with earlier experiments [22], addition of the combination of IL-17 and TNF-α stimulated the production of IL-6 relative to control (Fig 3; S3 Table). The comparison of the results without IL-17 and TNF-α addition showed that Zn addition enhanced IL-6 expression. The results were particularly demonstrative for OA synoviocytes, especially after 8 days (99.7 ± 25.3 ng/ml for OA cells treated with 3.6 ppm of Zn, vs. 33.0 ± 9.2 ng/ml without). The presence of IL-17 and TNF-α made the effect of Zn addition more difficult to interpret for OA cells, while the effect was clear for RA cells. In RA synoviocytes, both the combination of IL-17 and TNF-α and 3.6 ppm of Zn induced the highest level of IL-6, with Zn amplifying the effect of the added cytokines (106.4 ± 15.65 ng/ml, with cytokines only vs. 237.4 ± 35.58 ng/ml with both cytokines and Zn, p<0.01).

### A model of cytokine-induced zinc homeostasis regulation

In the presence of pro-inflammatory cytokines, Zn uptake was enhanced in synoviocytes as consequence of the increased expression of the ZIP-8 importer relative to the lack of change in ZnT1 exporter gene expression. We therefore analyzed the results of Zn changes in synoviocytes and the modulation by inflammation signals, through a simple transport model. Quantitative models of cellular Zn homeostasis have been previously proposed by Colvin et al. [23,24], but the number of analyzed parameters remains very large. Using the variables studied here in vitro, we have set up a simple model of Zn transport, in which the respective roles of cytokines and Zn concentration were taken into account.

**Fig 4:**
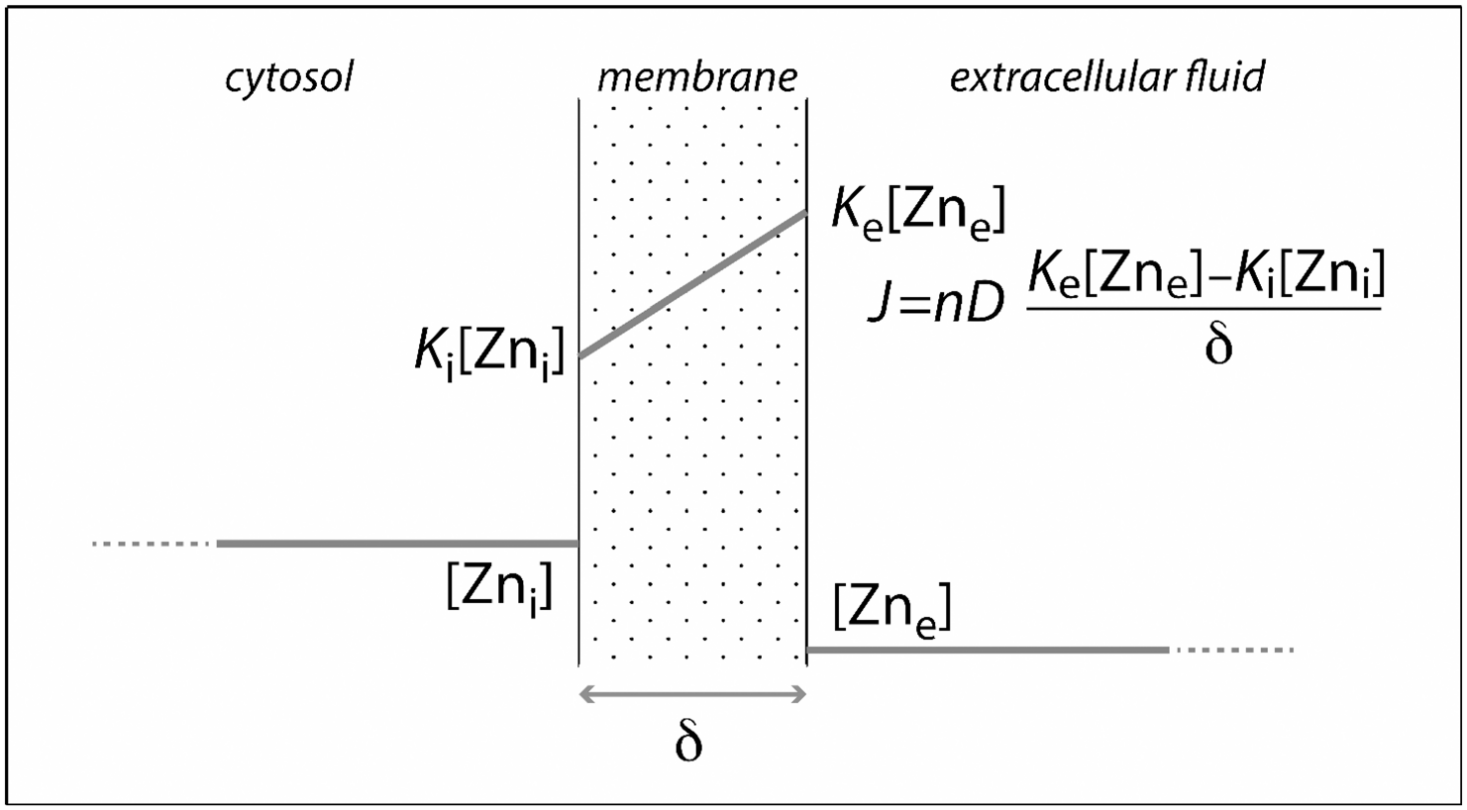
Model of diffusion-controlled Zn transport across the cellular membrane. A schematic model of Zn transport in cells takes into account the role of cytokines and Zn concentration. Fick equation describes the flux. *J*: total Zinc flux in cells through transporters; *n*: number of transporters; *D*: diffusion coefficient; *k*: partition coefficient, proportional to Zn concentration, where *e* and *i* stand for external and internal compartment respectively; *δ*: membrane thickness.

The diffusion-controlled model represents a cell as an inner cytosolic fluid separated from the extra-cellular medium by a membrane (Fig 4). Here, the number of Zn importer and exporter channels per cell is characterized by a single number, *n* for ZIP-8, and *n’* for ZnT1, respectively. Based on the K_D_ determination, we assume a steady-state transfer by diffusion across the membrane, which has a thickness of δ. The respective diffusion coefficients for Zn transfer are *D* and *D’*. We further assume that the proportion of occupied transporters, inside and outside the membrane, is proportional to the intra- and extra-cellular Zn concentrations. The proportionality constant is equivalent to a partition coefficient *K*. The total Zn fluxes *J* and *J’* across the transporters, can now be represented by Fick equations:

$$\begin{array}{l} J=nD\frac{K_{e}{\text{[Zn}}_{e}^{2+}\text{]-}K_{i}{\text{[Zn}}_{i}^{2+}\text{]}}{\delta }\\ J\prime =n\prime D\prime \frac{K\prime_{i}{\text{[Zn}}_{i}^{2+}\text{]-}K\prime_{e}{\text{[Zn}}_{e}^{2+}\text{]}}{\delta } \end{array}$$

in which subscript ‘*e*’ stands for the extra-cellular, and ‘*i*’ for the intra-cellular compartments. Non-primed and primed symbols refer to ZIP-8 and ZnT1, respectively.

Results on concentrations and isotopic abundance can be used to support the assumption that steady state was achieved very rapidly and that Zn is already linked to methionine, also at steady state. Therefore *J* and *J’* are equal. The two previous equations can be recast into:

$${\text{[Zn}}_{i}^{2+}\text{]}=\frac{nDK_{e}+n\prime D\prime K\prime_{e}}{nDK_{i}+n\prime D\prime K\prime_{i}}{\text{[Zn}}_{e}^{2+}\text{]}$$

In synoviocytes, the effect of IL-17 and TNF-α was largely independent of the Zn concentration. Since the *K* values reflect a difference in thermodynamic potentials and *D* values potential gradients across the membrane, the present results indicate that cytokines affect the number of channels *n* and *n’*. They indicate that *n* is enhanced independently of Zn concentration [5]. Since *n* is affected by cytokines to a greater extent than *n’*, the cell is primed by cytokines to control the Zn concentration level in the extracellular fluid.

The small overexpression of ZnT1 by cytokines and its dependence on Zn concentration indicates that the excess of Zn taken up by the cell to be stored by metallothioneins will eventually be excreted and the excretion rate of Zn will be slower than the uptake, leading to final intra-cellular accumulation.

Zinc residence time τ_Zn_ within a cell of volume *V* is:

$$\tau_{Zn}=\frac{V\left[Zn_{i}^{2+}\right]}{J^{\prime }}=\frac{\delta V}{n\prime \left(\left[Zn_{e}^{2+}\right]\right)D\prime \left(K\prime_{i}-\left(K\prime_{e}\left[Zn_{e}^{2+}\right]\right)\right)/\left(\left[Zn_{i}^{2+}\right]\right)}$$

where *n’* (ZnT1) is dependent on Zn concentration. The cell controls the concentration gradient between the intra- and the extra-cellular compartment by enhancing Zn export in response to a large excess of cytosolic Zn, which could induce toxicity.

Zn excess inside cells is stored in cytosolic metallothionein. Mammal metallothionein consists of two domains: a α-domain sequestering three Zn^2+^ ions, and an β-domain sequestering four Zn^2+^ ions [25]. The strong dependence of metallothionein expression reflects Zn storage requirements. The maximum Zn storage capacity by metallothionein is expressed as a sum of free Zn^2+^ and of the different complexes made with apo-metallothionein, i.e.,

$$\left[Zn_{total}\right]=\left[Zn_{i}^{2+}\right]+\beta_{1}\left[MT\right]\left[Zn_{i}^{2+}\right]+\beta_{2}\left[MT\right]{\left[Zn_{i}^{2+}\right]}^{2}+\ldots$$

where [MT] stands for the apo-metallothionein concentration in the cytosol and the β-values represent the stability constants of the Zn_*i*_ MT compounds (*i* = 1 to 7). Free Zn ions in the cytosol should be negligible [25]. Zn-excess in the extracellular medium therefore requires massive production of metallothionein, which is reflected by the Zn-dependent expression of the MT1 genes.

## Discussion

**Fig 5:**
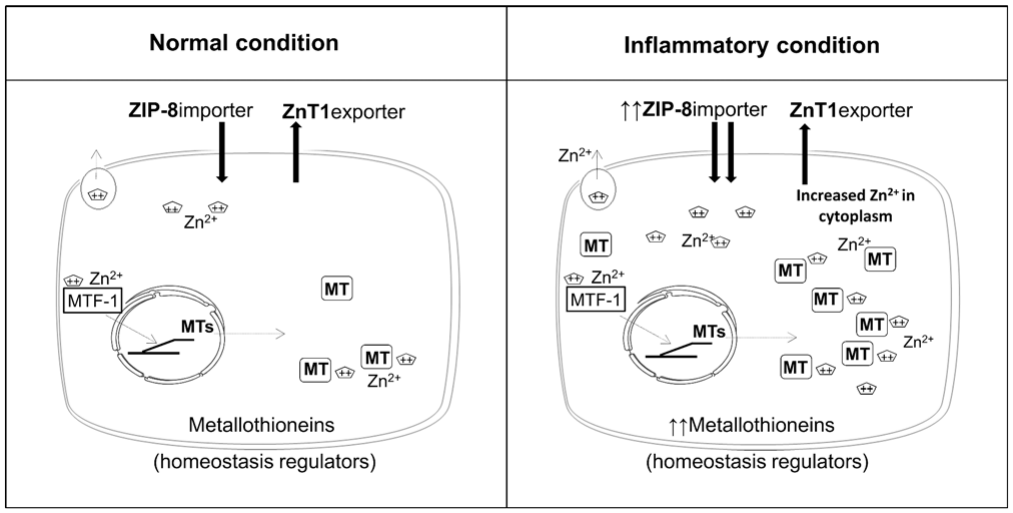
Zn transport in mesenchymal cells with and without inflammation. A simplified schematic view of Zn transport in mesenchymal cells. Arrows from cytoplasmic space to intracellular space indicate Zn entry by the ZIP-8 importer. Arrows from intracellular space to cytoplasmic space indicate Zn exit mediated by the ZnT1 exporter. MTs are transcribed via MTF-1 and MTs are necessary for Zn homeostasis in the cell. Non-inflammatory condition on the left panel is compared to inflammatory condition on the right panel. The picture shows an increase of ZIP-8 importer and of MTs during inflammation, correlating with an increased quantity of Zn inside the cell.

The combination of the in vitro results and their mathematical analysis shows that the pro-inflammatory cytokines IL-17 and TNF-α over-express Zn transporters, leading to a net export/import ratio in favor of Zn accumulation inside the cell (Fig 5). This concept seems sufficiently general to be applicable to other cell types. Our results suggest that both the up-regulated expression of the importer ZIP-8 and of the homeostasis regulators MTs, in the presence of inflammation, contribute to Zn accumulation in cells (Fig 5). Other studies support our results of inflammation triggering Zn-regulator gene expression and Zn accumulation in human cells [26,27]. This may lead to the discovery of new therapeutic options, targeting Zn-binding groups [28] to control inflammation [29].

An important consequence is the induction of inflammation associated with the accumulation of Zn inside cells. We focused on the production of IL-6, the main pro-inflammatory cytokine produced by synoviocytes. The effect of Zn on IL-6 production indicated a feedback loop, where the production of IL-17 and TNF-α upon original inflammation increases Zn uptake, leading to excess accumulation of Zn that promotes IL-6 production. Such self-sustained inflammation therefore has the potential of triggering chronicity. Interestingly, IL-17 and TNF-α stimulation of IL-6 production was faster for RA synoviocytes, but higher for OA than RA synoviocytes at the late stage. These results confirm that RA synoviocytes are early-responders and can easily adapt to the inflammatory stimuli. The use of OA cells as control for RA mainly rests on the notion that OA represents a non-/lower-inflammatory state [30]. No significant difference for Zn absorption was found between OA and RA synoviocytes, rather representing a continuum [30]. Further investigations should address the differences between OA and RA synoviocytes regarding the kinetics of Zn uptake and the factors defining the maximum Zn uptake by each type of cell.

Even if no change in Zn absorption was observed between the two cell types, Zn-induced mechanisms were different. ZIP-8 gene expression was increased by the IL-17 and TNF-α combination in both cell types. The combination of IL-17 and TNF-α was necessary for the increased expression of ZIP-8 in RA but not in OA synoviocytes. This results from a sequential effect where IL-17 primes RA synoviocytes to respond to TNF-α by enhancing the expression of the TNF-α receptor TNFRII [31]. MTs isoforms were less expressed in RA than in OA synoviocytes and their expression was sensitive to inflammation. MTs expression was enhanced by Zn alone in OA more than in RA synoviocytes,

In conclusion, independently of the cell type, inflammation from IL-17 and TNF-α combination plays a major role in altering Zn homeostasis in cells, even in the absence of added Zn. The changes in Zn homeostasis induced by inflammation then contribute to its self–activation and may play a strong role in the transition to the chronic stage of the disease.

In simple terms, the mathematical model expresses that inflammation opens the gates for Zn uptake and prepares the cell for Zn storage, regardless of the amount of Zn actually present in the extracellular medium. In contrast, inflammation does not efficiently prepare the cell for rapid excretion of metal excess. The price of increased cytokine production may signal the potential intoxication of inflamed tissues if metal levels in the extra-cellular medium become too high.

The same conclusion may apply to other metals of the Zn family (including some heavy metal as cadmium and mercury) and mimic Zn kinetics at the cellular level, accumulating in the cells linked to MTs. These results with Zn highlight the potential influence of the environmental factors associated with metals on the induction and progression of inflammation.

## Supporting Information

S1 Table Data table of zinc quantification in media and cells after exposure or not to IL-17 and TNF-α combination. (XLSX)

S2 Table Data table of transporter gene expressions after exposure or not to Zn and TNF-α and IL-17. (XLSX)

S3 Table Data table of IL-6 production in RA synoviocytes after exposure to Zn and pro-inflammatory cytokines. (XLSX)
